# Buried Alive: The Behavioural Response of the Mussels, *Modiolus modiolus *and *Mytilus edulis *to Sudden Burial by Sediment

**DOI:** 10.1371/journal.pone.0151471

**Published:** 2016-03-16

**Authors:** Zoë L. Hutchison, Vicki J. Hendrick, Michael T. Burrows, Ben Wilson, Kim S. Last

**Affiliations:** 1 Centre for Offshore Renewable Energy Engineering, Cranfield University, Cranfield, MK43 0AL, United Kingdom; 2 Department of Ecology, Scottish Association for Marine Science (SAMS), Oban, PA371QA, United Kingdom; Bangor University, UNITED KINGDOM

## Abstract

Sedimentation in the sea occurs through natural processes, such as wave and tidal action, which can be exacerbated during storms and floods. Changes in terrestrial land use, marine aggregate extraction, dredging, drilling and mining are known to result in substantial sediment deposition. Research suggests that deposition will also occur due to the modern development of marine renewable energy. The response to individual burial under three depths of sediment, three sediment fractions and five burial durations was investigated in two mussel species, *Modiolus modiolus* and *Mytilus edulis* in specialist mesocosms. Both mussel species showed substantial mortality, which increased with duration of burial and burial by finer sediment fractions. *M*. *modiolus* was better able to survive short periods of burial than *M*. *edulis*, but at longer durations mortality was more pronounced. No mortality was observed in *M*. *modiolus* in burial durations of eight days or less but by 16 days of burial, over 50% cumulative mortality occurred. Under variable temperature regimes, *M*. *edulis* mortality increased from 20% at 8°C to over 60% at 14.5 and 20°C. Only *M*. *edulis* was able to emerge from burial, facilitated by increased byssus production, laid mostly on vertical surfaces but also on sediment particles. Emergence was higher from coarse sediment and shallow burials. Byssus production in *M*. *edulis* was not related to the condition index of the mussels. Results suggest that even marginal burial would result in mortality and be more pronounced in warm summer periods. Our results suggest that in the event of burial, adult *M*. *modiolus* would not be able to emerge from burial unless local hydrodynamics assist, whereas a small proportion of *M*. *edulis* may regain contact with the sediment water interface. The physiological stress resulting in mortality, contribution of local hydrodynamics to survival and other ecological pressures such as mussels existing in aggregations, are discussed.

## Introduction

Benthic marine organisms, particularly filter feeders in near shore habitats, are adapted to surviving in environments that are usually rich in suspended particulate matter (SPM) [1]. The SPM provides an organic/inorganic mix of essential food, as well as resources for building dwelling tubes, habitat provision and camouflage. Levels of SPM are influenced by terrestrial input, seabed type, waves, currents and tides and will therefore vary naturally from site to site. Any reduction in water flow will result in deposition of SPM (sedimentation) and potentially bury infaunal, and sessile or slow moving epibenthic organisms [2]. Such smothering can be of natural and/or anthropogenic origin. Surviving a smothering event depends on whether an organism can survive burial or escape by re-emerging at the sediment surface. Mortality in mussels due to burial by sand has been documented [3, 4] yet survival and the ability to emerge from burial are poorly understood in *Modiolus modiolus* and *Mytilus edulis* and form the basis of this study.

Large changes in sedimentation can be the result of natural causes such as storms, floods and extreme tides but sedimentation increasingly occurs as a result of human activity, including alterations in terrestrial land use and beach restoration [5]. Traditional marine industries are a major source of sedimentation. Over 15 million tonnes of marine aggregate was extracted in Europe in 2010 for construction, beach nourishment and coastal defence [6]. Overspill and rejected material, which can be several tonnes per day, results in substantial sediment suspension and deposition [7–9]. Dredging to maintain access to UK fishing ports and harbours results in large volumes of material disposed at designated sites each year [10–12]. In addition to the physical challenge of increased SPM, sedimentation from industry may also carry high levels of metals and contaminants. For example, mining for metals can release tonnes of marine deposits with known contaminants [13, 14]. Additionally, drill cuttings from oil and gas installations contain toxic contaminants, even where modern water-based drill techniques are used [15].

More recently, the development of marine renewable energy technology has seen installations of offshore wind, wave and tidal energy devices having varying effects on the seabed, sometimes mobilising thousands of cubic meters of sediments [16]. Models of the effects of tidal energy extraction suggest changes in current flows and wave dissipation over regional scales [17]. The effects on sedimentary processes could be substantial [18–20]. For example, seabed height and sediment type may change several kilometres from a wave or tidal array [19–21]. It is also possible that the relocation/loss of sandbanks and associated communities may occur and altered wave dissipation towards shores could further effect coastal protection [18, 22].

Both natural and anthropogenic sedimentation can alter the topography of the seabed and its height as well as properties of the sediment such as sediment grain size, trace metal contamination and the organic content [9, 23, 24]. Changes in benthic communities are commonly reported, with studies often focusing on changes to community structure and composition, fauna abundance, productivity, the presence/absence of trophic groups, increases in opportunistic species and decreases in biomass, density, and species richness and diversity [9]. Such changes occur at the site of impact but often stretch further afield, sometimes evident many kilometres from the site of origin [24]. Benthic recovery to a pre-disturbance level, can take years, if it occurs at all [25].

To date experimental burial studies have focused mostly on intertidal and some subtidal species, representing both infaunal and epibenthic species of several classes, for example, molluscs, crustaceans, ascidians, annelids [26], and corals [27]. Studies have used a range of depths of burial, from 0.5 to 85 cm, using singular and combined particle fractions ranging from silts to sands, and measured mortality caused by burial over durations ranging from 1–2 hours to 15 days [28]. Where species migrated vertically, the orientation, distance migrated and mechanism of migration was reported. Previous experimental work has shown responses of benthic organisms to be variable and species-specific [29]. Many factors influence the response to burial (e.g. sediment type, life history, habitat, trophic group, motility of the organism and the ability to vertically migrate) and collectively determine the outcome for a particular organism.

An early study of 25 species of bivalves, showed that epibenthic suspension-feeding species which attach to substrates via byssus threads had little potential to escape and were susceptible to smothering [30]. *M*. *modiolus* and *M*. *edulis* fall into this group and share similar life traits in that they are both: relatively sedentary with limited mobility; they are both epibenthic byssate species although *M*. *modiolus* can also be considered endobyssate and often occurs semi-infaunally or infaunally; and both species occur subtidally and intertidally [31–33]. Changes in sand levels resulting in burial have been reported as an important factor for the lower distribution limits of *M*. *edulis* in the intertidal zone [34]. However, it has been reported that *M*. *edulis* were capable of resurfacing when covered in sand but that large-scale movements of sand were likely to bury *M*. *edulis* beds [35]. Despite reports of only limited escape potential [30, 35], *M*. *edulis* could emerge from a 6 cm increase in sediment level, in 1 to 2 days, although the method of vertical movement was unclear [36]. The Marlin Sensitivity Index (MSI) provided a theoretical assessment of the response of *M*. *modiolus* [37] and *M*. *edulis* [33] to smothering. *M*. *modiolus* was considered to be less tolerant, with a lower recoverability, than *M*. *edulis*, based on the higher mobility and more reliable recruitment of *M*. *edulis* [35, 38]. The MSI [33, 37] suggests the overall sensitivity to smothering (5 cm, one month) for *M*. *modiolus* is ‘high’ but 'low' for *M*. *edulis*, although to date there is little evidence to support these conclusions [33, 37]. There is particular importance in understanding the sensitivity of *M*. *modiolus* since it has been suggested that the demise of the Strangford Lough *M*. *modiolus* beds may in part have been due to smothering by sediments disturbed during trawling activities [39–41].

*M*. *modiolus* and *M*. *edulis* beds are important habitats with many ecosystem functions that form on a range of substrates (muddy, sandy and mixed sediments with stones and shells [35]). For example, the habitat heterogeneity and structural complexity of the mussel beds provide refuge to other species and promote biodiversity [42, 43]. Both types of mussel beds can support numerous species although *M*. *modiolus* beds are known to have higher diversity, and are suspected nursery grounds for *Pecten maximus* and *Aequipecten opercularis* [35, 41, 42]. The mussels themselves and associated fauna often provide essential food sources to higher and commercially important organisms, as well as playing a role in the control of phytoplankton dynamics [35, 44]. The hard structures formed by mussel beds, increase seabed roughness and modify hydrodynamic flows encouraging sediment deposition and stabilisation [45] whilst the production of biodeposits, enrich the seabed and enhance nutrient cycles [35, 36, 46]. Mussel beds formed by these species are recognised as habitats under Annex I of the European Habitats Directive [47], and protected under many legislative facets [48]. In addition *M*. *edulis* is commercially valuable in the UK [49].

Their ecological and economic importance, coupled with the low confidence of the MSI [33, 37] warrants further investigation of the response of *M*. *modiolus* and *M*. *edulis* to sediment burial. While *M*. *modiolus* and *M*. *edulis* can occur in close proximity to the aforementioned industrial activities [42, 50], our understanding of their true response to burial is poor. There are conflicting/vague reports for *M*. *edulis* [30, 35, 36] and despite suggestions that *M*. *modiolus* are susceptible to smothering [39–41], there is a general lack of data to support this. Survival is likely to be increased if mussels are capable of emerging from burial, therefore it would be useful to determine how robust the emergence behaviour in *M*. *edulis* is, and if this is a shared characteristic with *M*. *modiolus*. Although *M*. *edulis* has higher motility than *M*. *modious*, the latter can form semi-infaunal and infaunal beds and has developed different phenotypes in response to environmental conditions [31, 35] therefore may be less susceptible to burial. Here, we will investigate the strategy for survival and behavioural responses of both species to sudden deposits of sediment under different sediment fractions and depths of burial, over variable durations and temperatures. This study aims to understand the basic contributing factors to mortality caused by burial and the behavioural strategies for survival.

## Methods

### Specimen collection

Adult *Modiolus modiolus* and *Mytilus edulis* were collected locally from Argyll on the west coast of Scotland, UK. No special permissions for these collections were required and animals were not sourced from designated reef habitat. *M*. *modiolus* were collected by a shellfish diver in the Sound of Kerrera (56.412° N, -5.489° W), whilst *M*. *edulis* were obtained from commercially grown stocks (Easdale Seafoods Ltd. (56.290° N, -5.612° W), and Inverlussa Shellfish Co. Ltd., Craignure (56.290° N, -5.612° W). Mussels were thoroughly cleaned of fouling species and separated from each other and debris. All specimens were acclimatised in the Alan Ansell Research Aquarium (SAMS) for at least a week, and in the experimental tanks for at least 36 hours prior to experimentation. Adult *M*. *modiolus* and *M*. *edulis* had average shell lengths of 10.8 cm (sd = 1.3, n = 270) and 5.2 cm (sd = 0.3, n = 640), respectively. Additionally, small *M*. *modiolus* ($\bar{x}$ = 5.1 cm, sd = 1.5, n = 13), were sourced by divers from Hadd Rock, Strangford Loch, Northern Ireland, UK (54.454° N, -5.593° W; WGS 84 datum).

### Experimental environment

All experiments were carried out in the research aquarium at SAMS during the autumn of 2010 and 2011. Experiments took place in nine Paddle Vortex Resuspension Tanks (pVoRTs) a modification of the those previously developed [51] by adding a central paddle which generates a current flow. All pVoRTs were supplied with a continuous flow of seawater (22 l h^-1^) sourced from sub-sand intakes in the Firth of Lorn, adjacent to SAMS laboratories. Seawater parameters were measured daily: dissolved oxygen (mean± SD: 11.0 ±0.3 mg/l), pH (8.2 ±0.01), salinity (30 ±1 PSU) and temperature (12.0 ±2°C, unless otherwise stated) for the duration of the experiments. Paddle rotation was set at 16 rpm (±0.5) providing a flow speed of 6–8 cm s^-1^ [29]. The nine pVoRTs and the specimen holding tanks were maintained under a 12 hour light—12 hour dark (12L:12D) photoperiod including 15 minute dawn and dusk dimming periods simulating ambient photoperiods at the time of the experiments.

### Experimental design

#### Assessing Mortality Under Burial–Experiment 1

**Fig 1:**
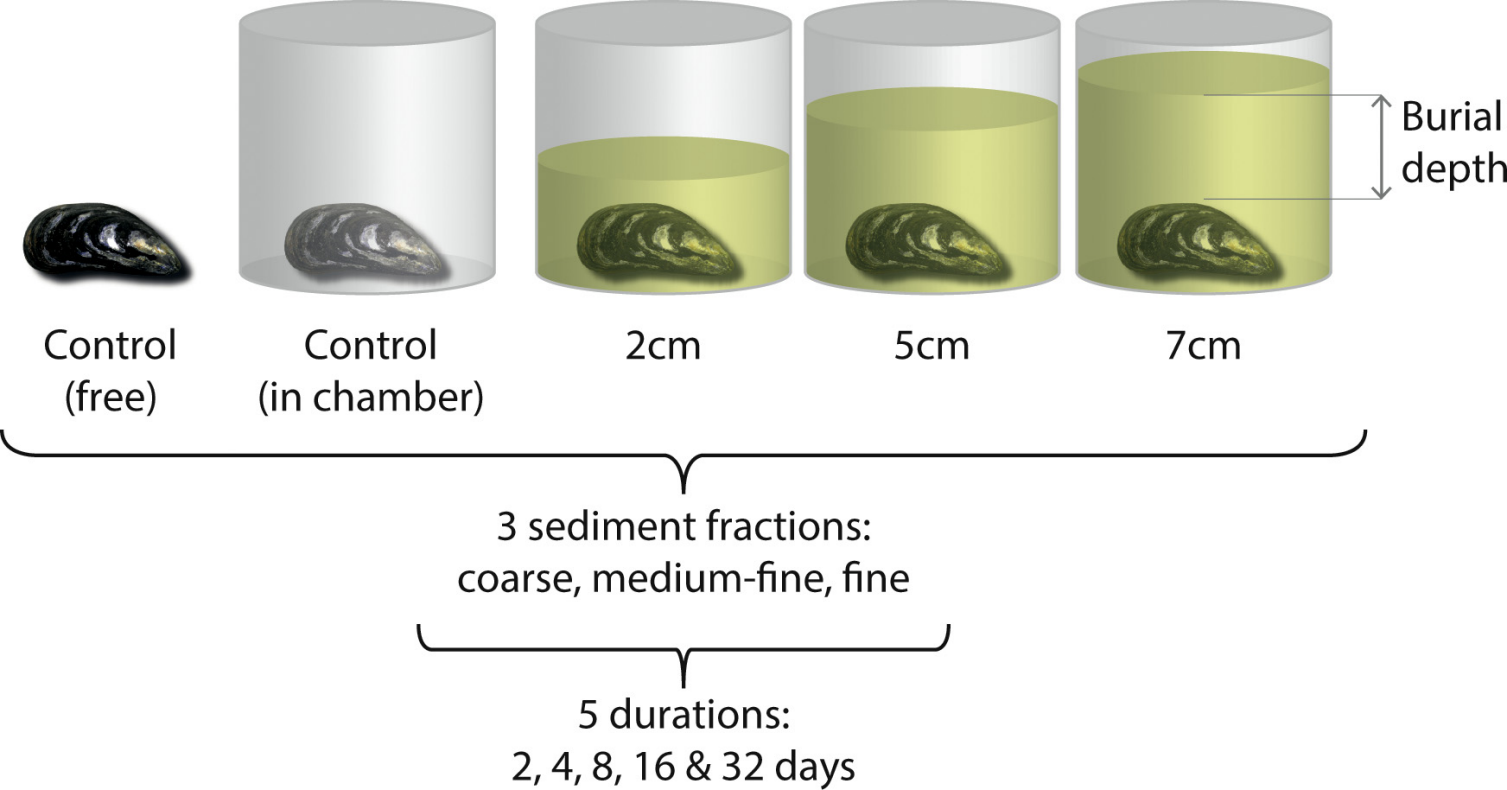
Schematic of multi-factorial nested experimental design. Each trial contained two control mussels (one free and one in a chamber) and three treatment mussels each burial to depths of 2, 5 and 7cm (n = 5, Control n (C_n_) = 2, Treatment n (T_n_) = 3). These five mussels were repeated for three sediment fractions; coarse, medium-fine and fine (n = 15, C_n_ = 6, T_n_ = 9) and for five burial durations (n = 75, C_n_ = 30, T_n_ = 45). Each set was replicated three times (n = 225, C_n_ = 90, T_n_ = 135), per species.”

A multifactorial experimental design, manipulating burial depth, sediment fraction and burial duration (after [29]) was used to assess mortality under sediment burial. The experiment incorporated three burial depths, shallow (2 cm), medium (5 cm) and deep (7 cm); three burial sediment fractions; coarse (1.2–2.0 mm diameter), medium-fine (0.25–0.95 mm) and fine (0.1–0.25 mm), and five burial durations of 2, 4, 8, 16 and 32 days. Sediment fractions were naturally washed, kiln dried, marine quartz obtained from Specialist Aggregates Ltd. The burial depths and sediment fractions were chosen based on the expected increase in sediment levels within 500 m out with the primary impact zone of marine aggregate and dredging activity [9, 29, 52]. Each burial depth, measured from the highest point of the bivalve, was repeated for each sediment fraction, with every combination of sediment fraction and depth repeated for each duration of burial (Fig 1). Two unburied control specimens were included in each trial, one where the mussel was placed in an experimental chamber and one which was placed freely on the mesh base of the pVoRTs to allow assessment of the effect of the chamber (Fig 1). Four hundred and fifty adult mussels were used in total (n = 225 per species) with each animal only assessed once at the end of the burial duration.

Experimental chambers were 180 mm diameter for *M*. *modiolus* and 76 mm in diameter for *M*. *edulis*, giving an approximately similar relationship between the volume of the mussels and the chamber. Mussels were in direct contact with the base of the burial chambers. Mussels were randomly assigned to a burial treatment, measured and placed on the bottom of the experimental chamber in a foot down orientation and buried according to the predefined burial treatment. The burial chambers were then carefully submerged and placed in the randomly assigned pVoRTs where they remained undisturbed for the duration of the burial. At the end of the burial period, emergence by the mussels was recorded if any part of the animal was visible at the sediment surface. All mussels, emerged or buried, were then removed from their chambers and survival was assessed by observing foot, shell gape and/or mantle movement. If no movement was evident then mortality was recorded.

#### The Influence of Temperature on M. edulis Mortality due to Burial–Experiment 2

To investigate whether the mortality observed in *M*. *edulis* was likely to vary with temperature, a further burial experiment was conducted at three temperatures representing the range likely to be experienced in UK coastal waters [53]. Specifically, the water in the pVoRTs were adjusted using a water cooler/heater (TECO®) to 8°C (± 1), 14.5°C (± 1), and 20°C (± 1), representing cold, ambient and warm water (n = 90). The warm water treatment is towards the upper thermal limit of distribution for *M*. *modiolus* (23°C) and *M*. *edulis* (27°C) [54] but not exceeding it. The burial experiment described previously was repeated using only fine sediment, since *M*. *edulis* was found to be most sensitive to burial by this sediment fraction (see Results: Mortality Due to Sediment Burial (Exp. 1)). Three burial depths were assessed; shallow (2 cm), medium (5 cm) and deep (7 cm) and only two durations, 16 and 32 days. Each animal was only assessed once at the end of each burial duration.

#### Investigating Emergence from Burial and Byssus Production–Experiment 3

Emergence in Small *M*. *modiolus* (Exp. 3a): Since emergence from burial is central to increasing the chance of survival, this behaviour was investigated in greater detail. Adult *M*. *modiolus* did not emerge from burial in Experiment 1 (see Results: Mortality Due to Sediment Burial (Exp. 1)), possibly due to the larger size, therefore small *M*. *modiolus* (n = 13) were assessed for emergence behaviour from shallow, coarse sediment burials for a single duration of 7 days.

Characterising Emergence Behaviour in *M*. *edulis* (Exp. 3b): In order to investigate whether increased byssus production was associated with the emergence from burial or an inherent behavioural trait, byssus threads were quantified prior to and after burial. Mussels were placed in the experimental chambers in the pVoRTs for 72 hours, after which the number of byssus plaques laid and the attachment point on the chamber (vertical or horizontal surface), were recorded for each mussel. Randomly, half of the mussels were removed, the chambers were cleaned of all byssus threads and plaques, and the animals returned to the same chamber. The remaining mussels were left attached to the chambers by their byssus threads. Randomisation was important to ensure that the mussels selected for burial or non-burial were not biased for byssus production. The burial experiments for *M*. *edulis* were repeated as outlined in Experiment 1 but using only coarse sediment and shallow, 2 cm burials for five durations; 1, 2, 4, 8 and 16 days (n = 90). Emergence events were recorded daily and after the designated duration of burial, the byssus plaque count and attachment point (vertical/horizontal surface, or on the sediment) was recorded for all mussels. Each animal was only assessed once at the end of the burial duration.

#### Byssus production and mussel condition–Experiment 4

To determine if byssus production was associated with mussel condition [55], *M*. *edulis* (n = 99) were placed in experimental chambers without sediment, in the pVoRTs. Byssus production (detailed in Experiment 3) was recorded after 72 hours and two indices of body condition calculated [56]; the gravitational condition index (dry soft tissue weight x 1000 / internal shell cavity capacity) to determine relative differences in nutritive status and the shell condition index (dry soft tissue weight x 1000 / dry shell weight) to detect differences in the absolute index of metabolism.

### Analysis

A binomial generalized linear model (using GLM in R version 2.15.1 [57]) with a logit link function was used to analyse the influence of burial depth, sediment fraction and (log transformed) burial duration on the probability of mortality under sudden burial (Experiment 1). The first model fitted tested whether the level of mortality in the buried mussels (n = 90) was significantly different to the mortality level in the control mussels (not buried, n = 135). This confirmed that the level of mortality in the treatment mussels (buried) was significantly higher than mortality in the control mussels (not buried) and therefore the control mussel data was excluded from further analysis. The control mussels were procedural controls only, to ensure that significant levels of mortality were not occurring due to an unknown factor affecting all mussels in the experimental environment. Data on mussels that remained buried at the end of Experiment 1 were included in subsequent analysis to determine which explanatory variables (burial depth, sediment fraction, burial duration and reasonable interactions) influenced mortality under sudden burial.

Responses of mussels in each treatment were then assessed by fitting models with all terms, both with and without interactions among variables. Interactions of the explanatory variables (burial depth, sediment fraction, duration of burial) were considered but interactions of the explanatory variables with mussel length were omitted, to reduce the risk of overfitting [58]. Non-significant explanatory variables and interactions were sequentially removed from the model using the drop1 function, returning the best fit model for the observed data set. The drop1 function used the chi-squared test to determine the significance of explanatory variables included in the scope of the specified model. Model selection using this function is based on the Akaike Information Criterion (AIC) [59], which identifies the best model to describe the mortality observed during the burial study. Predicted data were then used to plot the model, further confirming that the model was the best fit to the observed data. A binomial GLM with the logit link was also used to analyse the influence of the duration, depth of burial and water temperature on the probability of mortality while buried (Experiment 2).

Similarly, a binomial GLM with the logit link was used to analyse the influence of the duration of burial on the probability of emergence from sudden burial (Experiment 3). Since byssus production was initially stimulated by burial, we analysed the differences in numbers and locations of threads produced by buried and unburied mussels before and immediately after (1, 2 and 4 days) burial. Locations of byssus plaques (vertical and horizontal surfaces of the chamber, and the sediment grains) were analysed since these may influence emergence from burial and survival.

A linear model was used to investigate whether the byssus production was related to the condition of the mussels (Experiment 4). Length of the mussel was also included as an explanatory variable in this analysis.

## Results

All significant effects reported here were statistically significant at *p* = 0.05 or less unless otherwise stated.

### Mortality Due to Sediment Burial (Exp. 1)

No mussels died in non-buried controls (n = 90 per species) while a total of 21% of *M*. *modiolus* and 13% of *M*. *edulis* mussels (n = 135 per species) died in experimental treatments (binomial GLM: β = 18.24, *z* = 0.018, *P* = 0.986 and binomial GLM: β = 17.66, *z* = 0.017, *P* = 0.986, respectively, n = 225 per species). Data from non-buried control mussels were therefore excluded from subsequent analysis.

**Fig 2:**
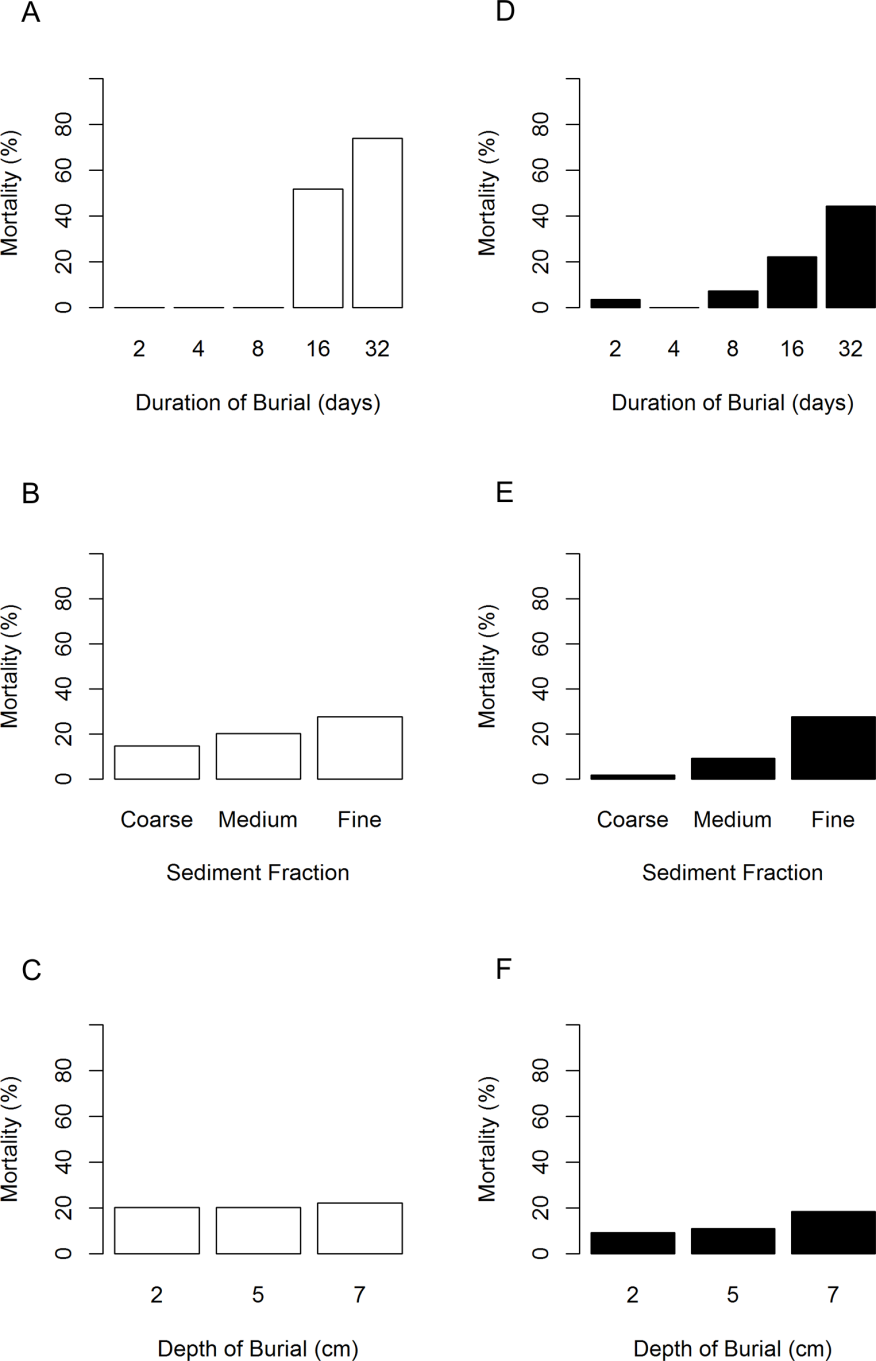
Mussel mortality influenced by duration, sediment fraction and depth of burial (Exp. 1). Mortality in *M*. *modiolus* under sediment burial of variable durations (A) sediment fractions (B) burial depths (C) and the same for *M*. *edulis* (D, E, F) in a multifactorial experiment to investigate mortality as a response to sudden episodic burial.

The probability of mortality in *M*. *modiolus* under burial significantly increased with increasing duration (S1 Table, Fig 2A), from nearly zero at 8 days to over 50% at 16 days of burial (n = 27 per duration, S1 Table, Fig 2A). Mortality in *M*. *modiolus* significantly increased from 15% in coarse sediments to 28% in fine sediments (n = 24 per sediment fraction, S1 Table, Fig 2B). A significant interaction was found between the duration of burial and the sediment fraction in that mortality increased with increasing duration and finer sediment fractions (S1 Table, Fig 2B). Mortality was not, however, influenced by burial depth (S1 Table, Fig 2C).

Similarly, the probability of mortality in *M*. *edulis* under burial significantly increased with increasing duration of burial with only 4% mortality after 2 days of burial increasing to 44% at 32 days of burial (n = 27 per duration, Fig 2D). Mortality in *M*. *edulis* also significantly increased with increasingly finer sediment fractions; 2% mortality in coarse sediments increasing to 28% mortality in fine sediments (n = 54 per species, Fig 2E). No interaction between duration of burial and the sediment fraction was detected (S1 Table). Mortality in *M*. *edulis* was not significantly influenced by the depth of burial (Fig 2F).

**Fig 3:**
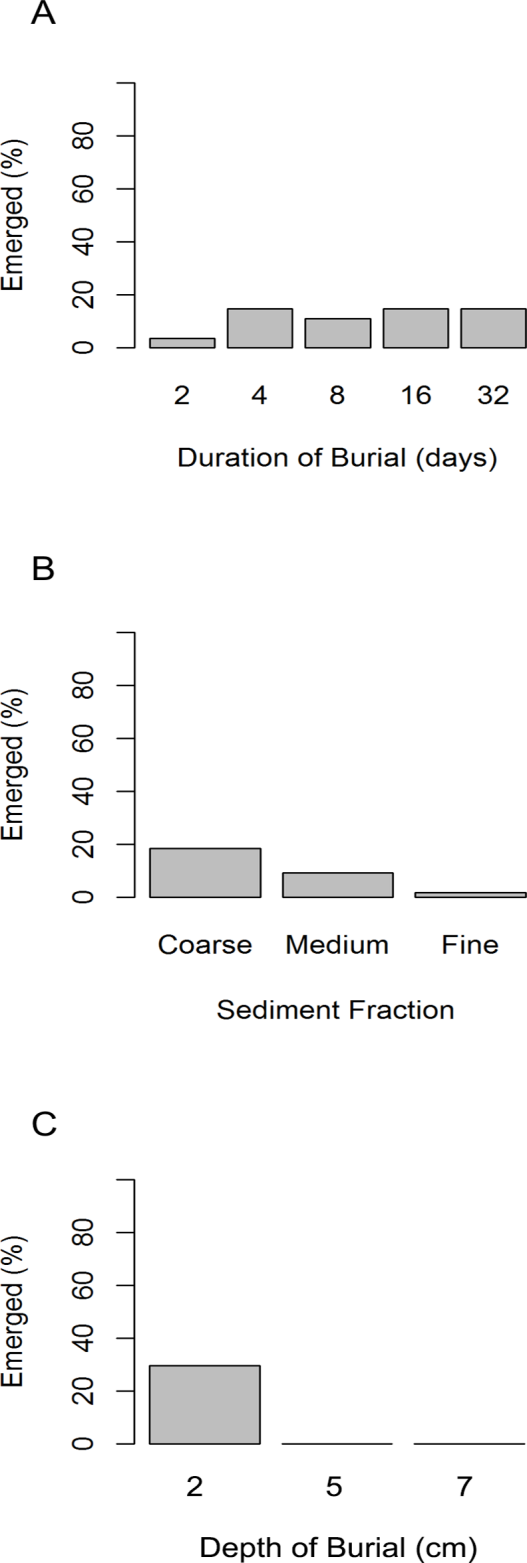
*M*. *edulis* emergence from burial (Exp. 1). The percentage of *M*. *edulis* which emerged from burial, in Experiment 1, under variable durations of burial (A), sediment fractions (B) and burial depths (C).

*M*. *edulis* were capable of emerging from burial, whereas *M*. *modiolus* was not. Of all *M*. *edulis* that were buried, 7% emerged (n = 135). Emergence was most evident in shallow burials (29%, n = 54) and coarse sediment (19%, n = 54, Fig 3).

### Influence of Temperature on Mortality of *M*. *edulis* under Burial (Exp. 2)

When sediment burial experiments were repeated under different temperatures with *M*. *edulis* 5.5% of non-buried control mussels died (n = 36) while a total of 55.5% of buried mussels (n = 54) died. The treatment (burial) was a significant factor in mussel mortality (binomial GLM β = 3.056, z = 3.3931, *P* = 8.45e-05). Data from non-burial control mussels were therefore excluded from subsequent analysis.

**Fig 4:**
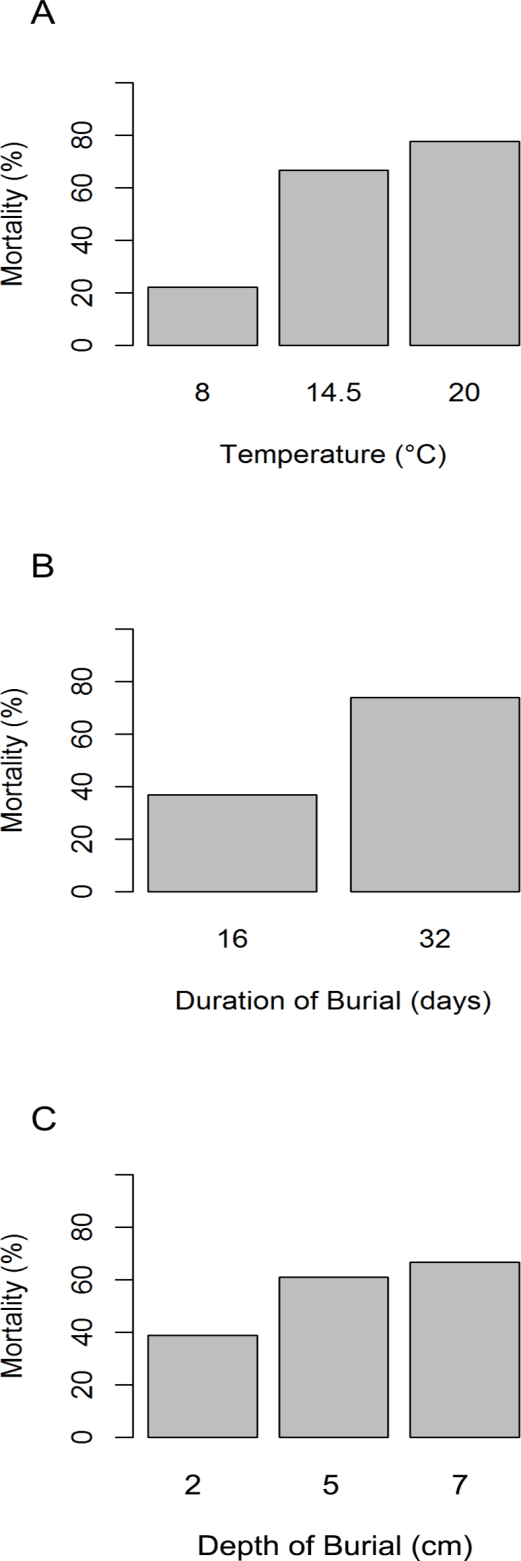
*M*. *edulis* mortality influenced by temperature under burial (Exp. 2). *M*. *edulis* were held under three water temperatures (A), two durations (B) and three burial depths (C) in a multifactorial experiment to investigate the variation in mortality as a response to sudden episodic burial across the thermal range of an annual seasonal cycle.

The probability of mortality significantly increased with increasing duration of burial, the depth of burial and the temperature of the seawater (Fig 4). Mortality increased from 20% at 8°C to over 60% at 14.5°C and 20°C (n = 18 per temperature). Furthermore a significant interaction between the duration of burial and the temperature of the seawater was found in that mortality increased with increasing duration of burial and seawater temperature (S2 Table).

### Emergence from Burial and Byssus Production (Exp. 3)

Emergence by Small *M*. *modiolus* (Exp. 3a): Emergence from burial was not observed in *M*. *modiolus* in Experiment 1. Since this lack of emergence may have been due to the difference in the size of the two species (adult *M*. *modiolus* are much larger than *M*. *edulis*), emergence in small *M*. *modiolus* was assessed. However small *M*. *modiolus* did not emerge from burial and only one of thirteen small *M*. *modiolus* showed vertical movement through the coarse sand but did not break the surface (data presentation not necessary).

Characterising Emergence Behaviour in *M*. *edulis* (Exp. 3b): Emergence behaviour observed in *M*. *edulis* (Exp. 1) was assessed, by repeating shallow burials in coarse sediment. There was no significant difference in levels of emergence between mussels that were attached by their byssus to the chamber or those unattached, prior to burial. Emergence from burial in each group was 62 and 64% respectively.

**Fig 5:**
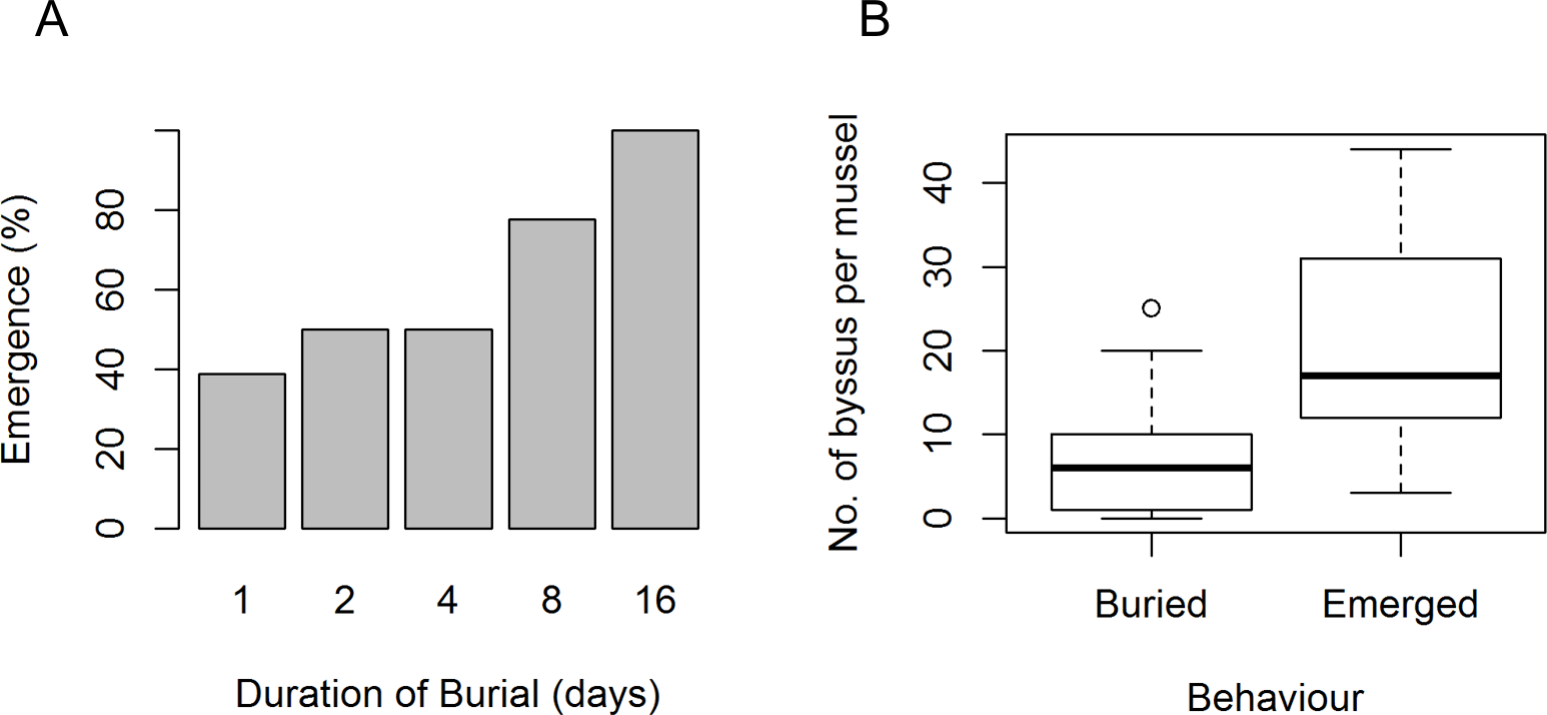
*M*. *edulis* emergence and byssus production (Exp. 3b). *M*. *edulis* emergence from shallow, coarse sediment burial over five durations (A). Emergence from burial was observed in 38% of mussels on day 1 and increased to 100% on day 16 (where n = 18, per duration). A higher degree of byssus production was observed in mussels that emerged (M = 17, range = 3–44, n = 57) than in mussels that remained buried (M = 6, range = 0–25, n = 33) (B).

**Fig 6:**
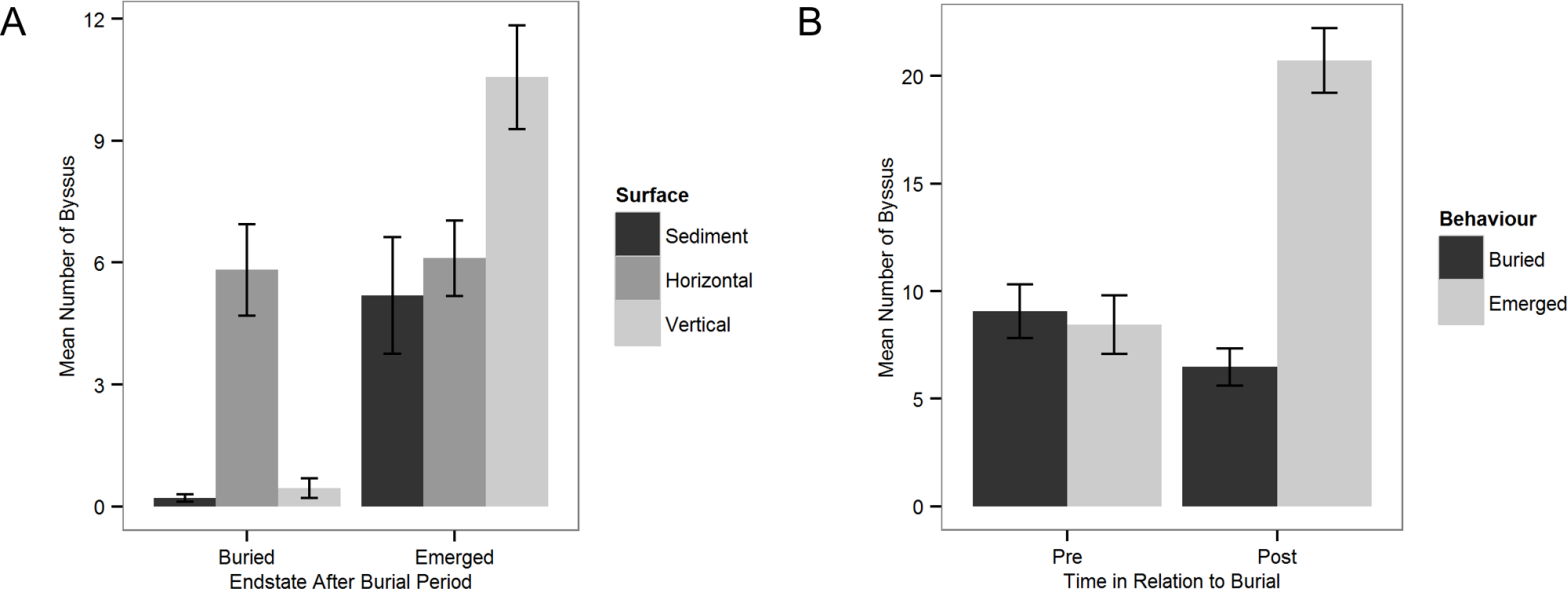
*M*. *edulis* byssus production on different surfaces and before/after burial (Exp. 3b). Counts of where the byssus threads were laid (A) indicated that there was higher activity on sediment particles (Buried sediment, x = 0.21, se = 0.09, emerged sediment x = 5.19, se = 1.44) and vertical surfaces (Buried vertical, x = 0.45, se = 0.24, emerged vertical x = 10.56, se = 1.28) in mussels which emerged than those that remained buried (remained buried n = 33, emerged n = 57). Byssus production in mussels prior to burial, measured after three days, was found to be similar (Buried, postburial x = 6.48, se = 0.87, Emerged, postburial x = 20.72, se = 1.51), but was higher for those that emerged, determined post burial (Buried, preburial x = 9.06, se = 1.25, Emerged, preburial, x = 8.44, se = 1.36)(B).

The probability of emergence from burial significantly increased with increasing duration of burial (Fig 5A, S3 Table), however in some cases emergence was observed within the first 24 hours. Total byssus production observed after burial was higher in emerged animals with an average 21 byssus threads (sd = 11, n = 57) compared to 6 byssus threads (sd = 6, n = 33) for those that remained buried (Fig 5B, S3 Table). We assessed the byssus production and attachment point to the surface of the experimental chamber (vertical and horizontal) and/or to the sediment during emergence. The number of byssus threads attached to vertical surfaces and to the coarse sediment were significantly higher in mussels that emerged than those that remained buried (Fig 6A, S3 Table). Mussels that emerged also produced more byssus than those that stayed buried (Fig 6B, S3 Table) indicating that byssus production is stimulated by burial.

### Byssus Production and Mussel Condition (Exp. 4)

**Fig 7:**
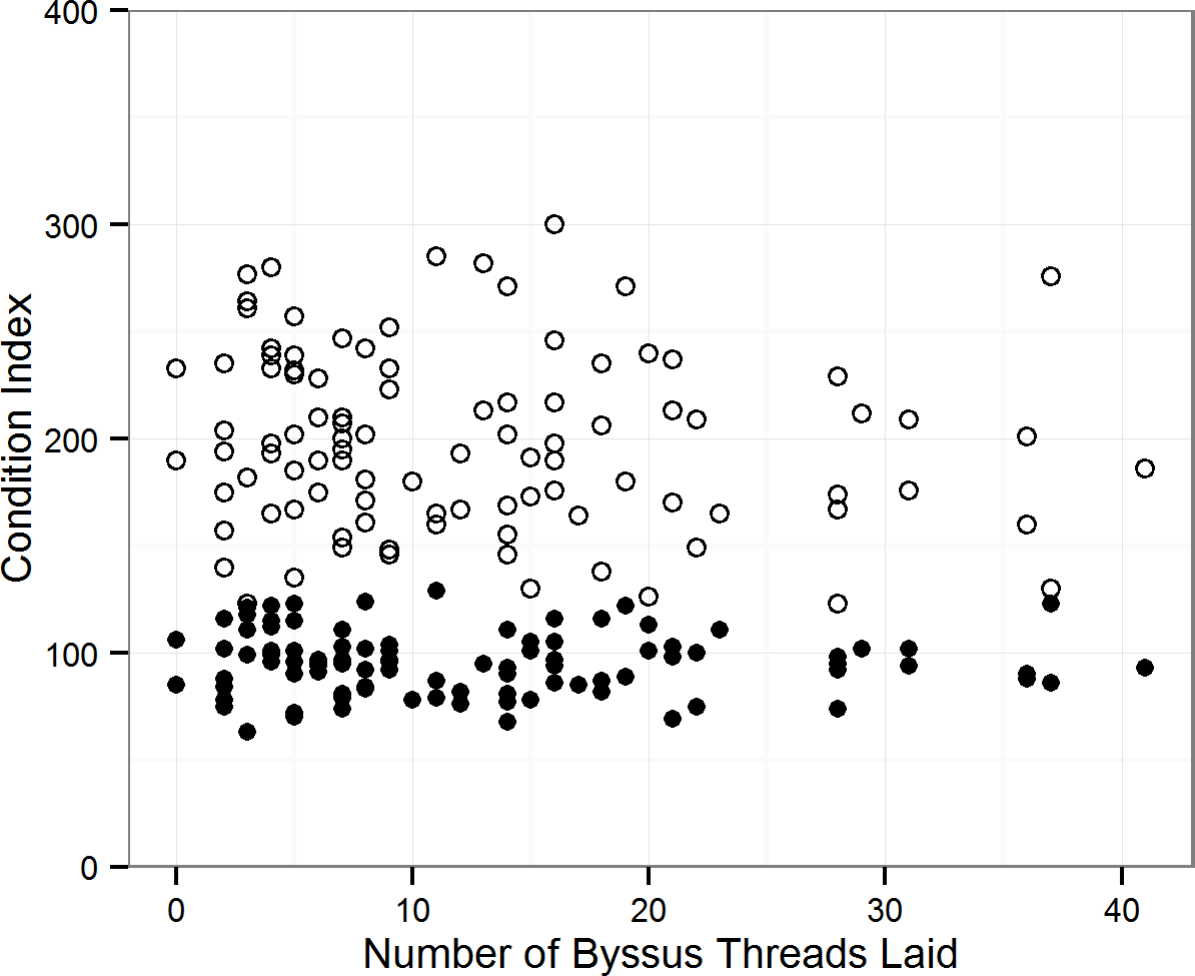
Byssus production and mussel condition (Exp. 4). The number of byssus threads laid by the mussels tested, was not affected by the condition of the mussel. This is demonstrated by the lack of a trend between the byssus number and the gravitational condition index (black) and the shell condition index (white).

The number of byssus threads laid by *M*. *edulis* was not related to mussel condition (adjusted R^2^ = 0.01653, *P* = 0.2069). The gravitational condition index had a range of 63–129 and the shell condition index, 123–300. Mussels generally produced between 0 and 20 byssus threads with fewer mussels laying between 20 and 41 byssus threads (Fig 7).

## Discussion

This study has shown that both *Modiolus modiolus* and *Mytilus edulis* were capable of surviving sediment burial in the short term (<16 days). However, with increasing burial duration (up to 32 days), mortality increased significantly, especially under finer-grained sediment. Whilst *M*. *modiolus* was more tolerant of short term burial (lower mortality under burial), *M*. *edulis* was more tolerant of long term burial partially due to its ability to emerge from shallow (<2 cm) burial. The emergence response in *M*. *edulis* was irrespective of body condition and relied on increased byssus production and the attachment of plaques, especially to vertical surfaces. Surprisingly, we did not observe an escape response in *M*. *modiolus*.

Behavioural and physiological laboratory studies usually use static water aquarium tanks which are not representative of the dynamic environment that marine organisms inhabit, especially suspension feeders that require water movement to supply food. This study was therefore facilitated by the use of pVoRT mesocosms that simulate natural water flow. Nevertheless, there are other natural factors that may influence mortality associated with increasing sediment load. For example, organic enrichment of natural sediments may reduce oxygen in pore water available to buried mussels [60]. While the nature of the sediment may modify responses, our experiments show simply how the physical nature of burial contributes to mortality in these bivalves.

*M*. *modiolus* and *M*. *edulis* share similar life traits in that they both have limited mobility and produce byssus threads to anchor themselves to hard and soft substrates and adjust their position by doing so. Despite these similarities, the species have quite different responses to sudden burial, the most apparent being that *M*. *edulis* emerged from burial whereas *M*. *modiolus* did not. The surprising lack of escape response by *M*. *modiolus* suggests that natural sedimentation events at established *M*. *modiolus* beds must be rare or gradual enough to avoid complete burial. It has however been suggested that the demise of the Strangford Lough *M*. *modiolus* bed may in part have been due to sediment disturbance by trawling activities [39–41]. Despite this, there are examples of semi-infaunal and infaunal *M*. *modiolus* beds in the UK [35, 41]. It is not recorded in the literature if *M*. *modiolus* allow themselves to become buried or actively bury themselves. No evidence has yet emerged whether, epibenthic, semi-infaunal and infaunal bed forming *M*. *modiolus* are the same genotype [35], however it is now known that these different ecotypes have different shell morphologies [31]. It is of note that infaunal beds tend to be in loose gravelly substrates [35] which is in keeping with the results of this study where mortality was lower in coarser sediment.

**Fig 8:**
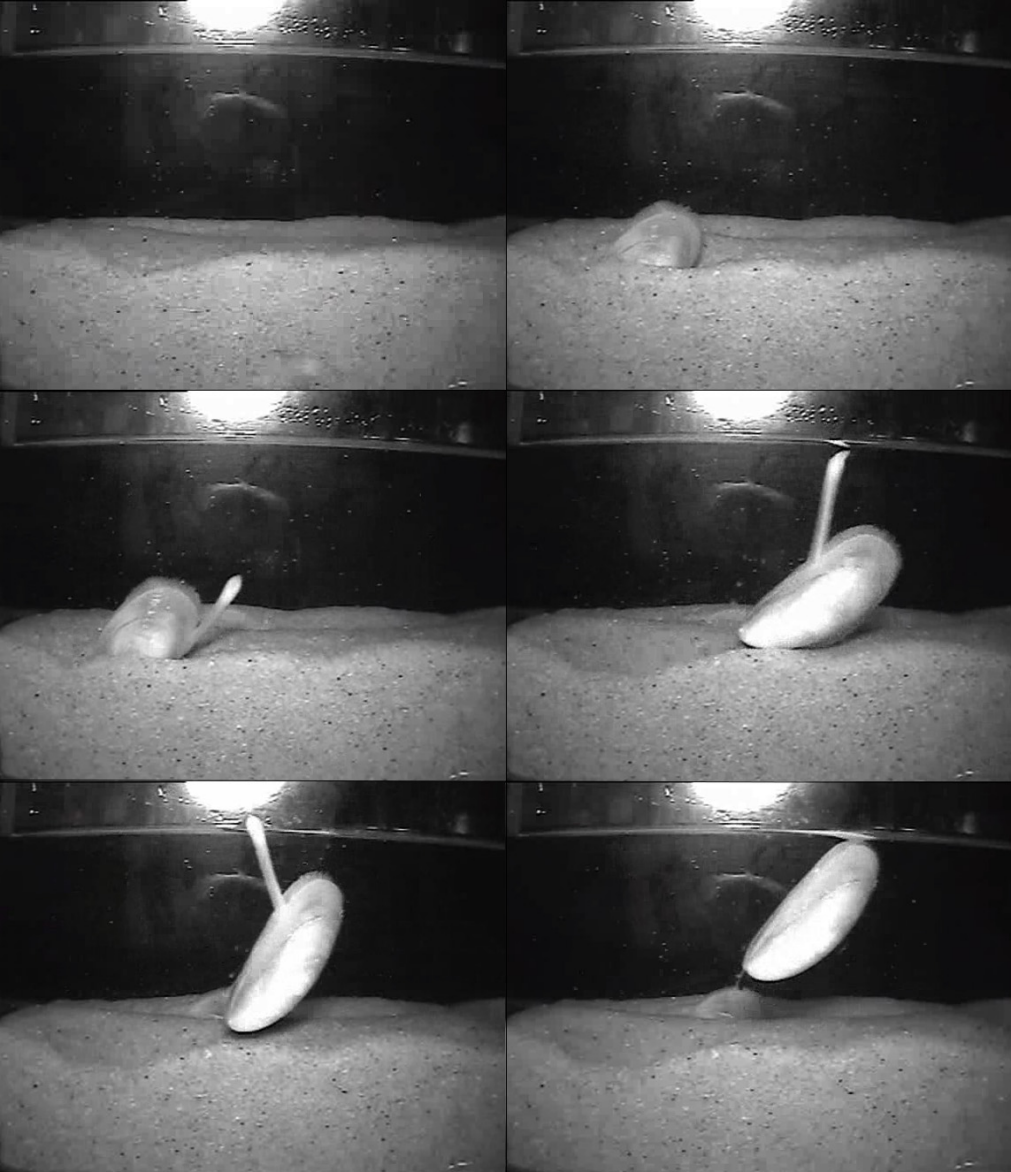
*M*. *edulis* emergence from burial. Still images from video footage (filmed under infra-red light) showing the emergence of *M*. *edulis* from sediment burial over the course of several hours at night. The foot was laying byssus threads on the sediment and on the vertical (glass) surface.

The availability of hard vertical surfaces (the side of the burial chamber) and coarse sediment particles were important for emergence from burial by *M*. *edulis* as demonstrated in Fig 8. In a bed, mussels are typically attached to each other to varying degrees by byssus threads [36]. Mussel beds with a higher density of individuals have a higher degree of byssal connections between mussels, whereas lower density beds have fewer connections, yet greater anchorage to the seabed [36]. Future research should consider the likelihood of emergence from burial of mussel beds with varying parameters such as density, porosity and structure. It is plausible that inter-mussel attachment may help mussels emerge from burial if buried individuals are attached to mussels higher in the bed. Equally, it may become a hindrance where mussels are strongly attached to many others. *M*. *edulis* have been observed to detach byssus threads [61], a finding which is in line with observations in this study where byssus were often broken or slackened (either by lengthening or stretching), and fresh threads attached at a higher point on the vertical surface. *M*. *modiolus* have not been observed to detach byssus threads and have been reported to have stiffer but less extensible byssus threads than *M*. *edulis* [62–64]. Comparative studies have shown that *M*. *modiolus* produces longer and more numerous byssus threads with larger plaques than *M*. *edulis* although both species increase the plaque size in response to larger particle size [62]. Despite this, *M*. *edulis* byssus are able to withstand greater force because the threads are thicker [63], which may be why they are able to be used to emerge from burial. Endobyssate species, including *M*. *modiolus* have been shown to exhibit a double yield in stress-strain assessments suggesting that endobyssate and epibyssate species likely produce physiologically and functionally diverse byssus threads [63–65]. Byssal attachment [66], growth and reproductive success have been shown to vary according to the spatial location of animals within mussel aggregations [67] and it seems likely that the spatial proximity of a mussel within a bed is likely to affect survival in the event of burial too.

Sediment deposition on a natural mussel bed will be different to the deposition in this study due to the inherent porous structure of the bed. This is exemplified in a laboratory study [36] where 6 cm of sediment was deposited on a medium density mussel bed in a flume. After one day, the mussel aggregation had restructured and sediment settled between the mussels resulting in the overall mussel aggregation being elevated and sediment level lowered. *M*. *edulis* were reported to be capable of emerging from 3 or 4 cm of burial [30], in keeping with data presented here since 3–4 cm of burial from the sediment water interface is likely to only be approximately 2 cm from the top of a large mussel, if horizontal. It was proposed that larger mussels of the same species are more capable of emerging since they have fewer body lengths to travel. Further work is required to determine if within a species, the size of a mussel influences the ability to emerge from burial, in particular with relation to juvenile mussels.

Our study supports previous observations that foot activity and byssal attachment to vertical substrates or surfaces are important in *M*. *edulis* for migration through sediments [30, 36]. Emerged mussels were typically in a vertical orientation (though originally horizontal when buried) supporting the hypothesis that the upper valve edge may assist in leverage [36]. The use of ventilation flows to fluidise sediment [30], although not observed, would seem likely since emergence was less evident in finer sediment and deeper burials. In these burials, the sediment would have been more difficult to fluidise, due to the compaction of the sediment combined with decreased ability to obtain (oxygenated) water through the sediment. The digging cycles of burrowing bivalves are well understood. Although *M*. *modiolus* and *M*. *edulis* are typically not morphologically or physiologically adapted for emerging from burial like specialist burrowing bivalves are for digging, the processes involved are likely to be similar. This includes a cyclic muscular control in probing the sediment, hydrostatic and haemolytic alterations and gape pattern [68] with the obvious difference being the byssogenesis. Additionally, it should be noted that the physical dimensions, which are important for digging [69] and may be equally important in emergence behaviour, are far greater in adult *M*. *modiolus* than in adult *M*. *edulis*. This difference may be a contributing factor in the lack of emergence in *M*. *modiolus* as well as the increased degree of emergence in coarser sediment for *M*. *edulis*, although smaller *M*. *modiolus* still showed no tendency to emerge.

It has been suggested that mussels may be able to detect the depth of overlying sediment since vertical migration slowed down as mussels approached the surface of the sediment [30]. While metabolic or muscular fatigue could also explain this slowing down of upward movement, it may also be related to the ease in which mussels are able to draw oxygenated water down through the sediment [30]. Considerable effort is required for a bivalve to turn whilst under sediment [30] and it is likely to be energetically costly to emerge. In addition to this, it is plausible that mussels under burial are experiencing hypoxic and/or anoxic conditions. Often when mussels were unburied in this study, black anoxic sediment surrounded the mussel. This could indicate that the mussels used the oxygenated water available within the sediment although it may also have been able to siphon water from the surface, more so in the coarser sediments where interstitial flow is facilitated. Some studies have indicated that *M*. *edulis* is tolerant of hypoxia and anoxia for short periods of time [70–72]. *M*. *edulis* may close their valves in response to hypoxia/anoxia and can switch proportionately from aerobic to anaerobic metabolism when required. The proportion of anaerobic metabolism, which is present even at normoxia, increases as oxygen falls below 90% saturation, however they also maximise oxygen consumption until oxygen levels fall below 60% saturation [73, 74]. During anaerobic metabolism *M*. *edulis* can reduce its energy demands to 5–20% of the aerobic energy demand ([75] and references therein) enabling survival in this state for up to 1–2 weeks [76] although survival after several weeks has also been reported [75].

Mortality in *M*. *modiolus* was not observed until the 16 day sampling point, although the level of mortality was high (>50%) suggesting that there was a threshold or tipping point between 8 and 16 days. There have been fewer studies on the hypoxic stress tolerances of *M*. *modiolus*, however it has been reported to be distinctly more tolerant than *M*. *edulis* under the dual pressures of reduced oxygen and presence of hydrogen sulphide [72]. Resistance to oxygen deficiency and elevated hydrogen sulphide is a result of reduced mechanical activity and metabolic activity [77]. This may explain why some *M*. *edulis* emerged whilst others did not, and why *M*. *modiolus* were more resistant to short durations of burial (immobility and subsequent reduced oxygen demand whilst under burial). After unburial, from prolonged burial (>16 days) and submergence in water, both *M*. *modiolus* and *M*. *edulis* were observed to gape widely which is most likely indicative of the significant oxygen debt characteristic of anaerobic metabolism, which must be paid off on return to aerobic metabolism [78]. It has been reported that oxygen deficiency in the presence of hydrogen sulphide was better endured at cooler temperatures [72]. Our study found higher mortality in *M*. *edulis* at higher seawater temperatures, most likely owing to an increased oxygen demand under higher temperatures. This suggests that burial occurring in the summer when seawater temperatures are higher may have potentially greater impacts, with mortality almost three times higher than that of winter seawater temperatures. There are also potential consequences for cumulative pressures of modern marine activities which cause sedimentation together with predictions of warming seawater. This may be of particular importance for *M*. *modiolus* which is a boreal species at the southern range of its distribution with predicted future habitat loss due to warming seawater temperatures [79].

Survival of *M*. *modiolus* and *M*. *edulis* under burial may be, to a lesser degree, influenced by other pressures. For example, another factor that may induce mortality whilst buried could be starvation, although this seems unlikely under relatively short burial durations since bivalves reduce filtration rates where algae are in low concentrations and alter their metabolism to accommodate this within 10–30 days dependent on the season [80, 81]. It is unlikely that mussels were attempting to feed whilst buried although if they were capable of respiring from interstitial water they may have benefited energetically from metabolising dissolved organic matter. Alternatively, if feeding was absent mussels may have utilised protein stores [80]. Nitrogenous waste from mussels, particularly if utilising proteins [80], may have accumulated in the sediment further polluting the burial environment. Additionally, mussels may have taken in sediment whilst under burial which would need to be eliminated. Dredged *M*. *edulis* rapidly discharged sediment within 15 minutes but took over 48 hours to complete elimination [82]. Energy budget absorbed by this process would reduce the energy available for normal metabolic processes, temporarily reducing their fitness.

Although remaining buried may not increase chance of survival in the long term, there may be benefits of not attempting to emerge from burial in the short term. Remaining buried may protect mussels from the effects of severe seabed disturbances, such as those from passing storms. Reduced mechanical and metabolic activity could substantially reduce scope for growth [83] and allow energy partitions to be redistributed aiding survival over a longer period of time. This is similar to responses to seasons, spawning and altered food availability [84–86]. Energy budgets could be replenished once the mussel is exposed by natural water movements, however this would be dependent on mussel density and current strength (see [36]) and more likely in spring/neap tidal cycle. The likelihood of unburial occurring is no doubt specific to the site since *M*. *edulis* and *M*. *modiolus* beds can be found in sheltered locations such as bays, fjords and lochs as well as moderately current swept locations [33, 37, 43]. Mussel beds with stronger currents will have a higher number of byssal attachments [36] which could render the probability of emergence reported in this study an underestimate. However, higher resuspension of sediments occurs in medium density mussel beds owing to higher turbidity and scouring at the edges of aggregations, which may assist mussels in emerging from burial. Reduced density of mussel beds (due to mortality) would increase the vulnerability of the bed to erosion, by high currents and storms [36]. Additionally, in these circumstances, isolation from the bed and/or reduced mussel condition may increase vulnerability to predation, [87].

The results of this study have demonstrated the effects of sediment type, depth of burial and duration of burial, as well as the temperature of the overlying water, as contributing factors to mortality in *M*. *modiolus* and *M*. *edulis* buried under suddenly deposited sediment. They have also revealed the strategies for survival of burial events, including emergence from burial by *M*. *edulis*. To better understand the burial responses, further research is required specifically to comprehend the influence of organic enrichment, pollutants, predator prey interactions, competitive behaviour, different life stages and hydrodynamics of mussel beds. Such information may aid the conservation of these important habitats and associated communities despite increasing industrial interest in the sea, changes in land use and expected changes in climate. Where anthropogenic sedimentation on a mussel bed is expected, targeted research should consider the species, particle size, depth of deposition and likely duration before granting licenses for various activities, as well as the current health of the mussel bed and hydrodynamics of the area.

## Supporting Information

S1 Table Experiment 1. Results of the best fit binomial GLM for the probability of mortality in *Modiolus modiolus* (a) and *Mytilus edulis* (b) whilst under burial of variable durations (2, 4, 8, 16, 32 days), sediment fractions (coarse, medium and fine) and depths (2 cm, 5 cm, 7 cm).
(DOCX)

S2 Table Experiment 2. Results of the best fit binomial GLM for the probability of mortality in *Mytilus edulis* whilst under fine sediment burial for variable depths (2 cm, 5 cm, 7 cm), durations (16, 32 days) of burial and variable water temperatures (cold (8°C), ambient (14.5°C) and warm (20°C) water).
(DOCX)

S3 Table Experiment 3. Results of the best fit binomial GLM for the probability of *Mytilus edulis* emerging from shallow, coarse sediment burial over increasing durations (a). It was confirmed that there was a higher level of byssus production in mussels that had emerged from burial (b). The use of the sediment, and hard surface in horizontal orientation was assessed and vertical surfaces were found to be important in the emergence behaviour (c).
(DOCX)

## References

[1] Miller DC, Bock MJ, Turner EJ. Deposit and suspension feeding in oscillatory flows and sediment fluxes. J Mar Res. 1992;50:489–520. 10.1357/002224092784797601

[2] Zhang W. Sediment dynamics 2014 In: Encyclopedia of Marine Geosciences [Internet]. Bremen, Germany: Springer Science Available: http://download.springer.com/static/pdf/940/chp%253A10.1007%252F978-94-007-6644-0_175-1.pdf?originUrl=http%3A%2F%2Flink.springer.com%2Freferenceworkentry%2F10.1007%2F978-94-007-6644-0_175-1&token2=exp=1436191032~acl=%2Fstatic%2Fpdf%2F940%2Fchp%25253A10.1007%25252F978-94-007-6644-0_175-1.pdf%3ForiginUrl%3Dhttp%253A%252F%252Flink.springer.com%252Freferenceworkentry%252F10.1007%252F978-94-007-6644-0_175-1*~hmac=b394e0289848263d04e8f452e1470b2863fbe0797579740200afdad16034fafe.

[3] Zardi GI, Nicastro KR, McQuaid CD, Erlandsson J. Sand and wave induced mortality in invasive (*Mytilus galloprovincialis*) and indigenous (*Perna perna)* mussels. Mar Biol. 2008;153(5):853–8. 10.1007/s00227-007-0857-z

[4] Zardi GI, Nicastro KR, Porri F, McQuaid CD. Sand stress as a non-determinant of habitat segregation of indigenous (*Perna perna*) and invasive (*Mytilus galloprovincialis*) mussels in South Africa. Mar Biol. 2006;148(5):1031–8. 10.1007/s00227-005-0155-6

[5] Thrush SF, Hewitt JE, Cummings VJ, Ellis JI, Hatton C, Lohrer A, et al Muddy waters: elevating sediment input to coastal and estuarine habitats. Front Ecol Environ. 2004;2(6):299–306. 10.1890/1540-9295(2004)002[0299:mwesit]2.0.co;2

[6] BMAPA. British Marine Aggregate Producers Association: Key Facts 2010 [cited 2015]. Available: http://www.bmapa.org/about/key_facts.php.

[7] Newell RC, Hitchcock DR, Seiderer LJ. Organic enrichment associated with outwash from marine aggregates dredging: A probable explanation for surface sheens and enhanced benthic production in the vicinity of dredging operations. Mar Pollut Bull. 1999;38(9):809–18. .

[8] HELCOM. Marine sediment extraction in the Baltic Sea—Status Report. Helsinki Comission, Baltic Marine Environment Protection Commission, 1999 Contract No.: No 76.

[9] Newell CR, Seiderer LJ, Hitchcock DR. The impact of dredging works in coastal waters: A review of the sensitivity to disturbance and subsequent recovery of biological resources on the seabed. Oceanogr Mar Biol Annu Rev. 1998;36:127–78.

[10] Boyd SE, Rees HL, Richardson CA. Nematodes as Sensitive Indicators of Change at Dredged Material Disposal Sites. Estuar Coast Shelf Sci. 2000;51(6):805–19. 10.1006/ecss.2000.0722.

[11] Essink K. Ecological effects of dumping of dredged sediments: options for management. J Coast Conservation. 1999;5:69–0.

[12] Wilber DH, Clarke DG, Rees SI. Responses of benthic macroinvertebrates to thin-layer disposal of dredged material in Mississippi Sound, USA. Mar Pollut Bull. 2007;54(1):42–52. 10.1016/j.marpolbul.2006.08.042. 17052734

[13] Castilla JC, Nealler E. Marine environmental impact due to mining activities of El Salvador copper mine, Chile. Mar Pollut Bull. 1978;9(3):67–70. 10.1016/0025-326X(78)90451-4.

[14] Olsgard F, Hasle JR. Impact of waste from titanium mining on benthic fauna. J Exp Mar Biol Ecol. 1993;172(1–2):185–213. 10.1016/0022-0981(93)90097-8.

[15] Trannum HC, Nilsson HC, Shaanning MT, Øxnevad S. Effects of sedimentation from water-based drill cuttings and natural sediment on benthic macrofaunal community structure and ecosystem processes. J Exp Mar Biol Ecol. 2010;383:111–21.

[16] Miller RG, Hutchison ZL, Macleod AK, Burrows MT, Cook EJ, Last KS, et al Marine renewable energy development: assessing the Benthic Footprint at multiple scales. Front Ecol Environ. 2013;11(8):433–40. 10.1890/120089

[17] Shields MA, Woolf DK, Grist EPM, Kerr SA, Jackson AC, Harris RE, et al Marine renewable energy: The ecological implications of altering the hydrodynamics of the marine environment. Ocean Coast Manage. 2011;54(1):2–9. 10.1016/j.ocecoaman.2010.10.036

[18] Neill SP, Jordan JR, Couch SJ. Impact of tidal energy converter (TEC) arrays on the dynamics of headland sand banks. Renew Energ. 2012;37:387–97.

[19] Neill SP, Litt EJ, Couch SJ, Davies AG. The impact of tidal stream turbines on large scale sediment dynamics. Renew Energ. 2009;34:2803–12.

[20] Walkington I, Burrows R. Modelling tidal stream power potential. Appl Ocean Res. 2009;31(4):239–45. 10.1016/j.apor.2009.10.007

[21] Jones C, Magalen J, Roberts JM. Wave energy converter (WEC) array effects on wave, current, and sediment circulation: Monteray Bay, CA California: Sandia National Laboratories for the United States Department of Energy, 2014.

[22] Abanades J, Greaves D, Iglesias G. Coastal defence using wave farms: The role of farm-to-coast distance. Renew Energ. 2015;75:572–82. 10.1016/j.renene.2014.10.048.

[23] Trembanis A, DuVal C, Beaudoin J, Schmidt V, Miller D, Mayer L. A detailed seabed signature from Hurricane Sandy revealed in bedforms and scour. Geochemistry, Geophysics, Geosystems. 2013;14(10):4334–40. 10.1002/ggge.20260

[24] Stronkhorst J, Ariese F, van Hattum B, Postma JF, de Kluijver M, Den Besten PJ, et al Environmental impact and recovery at two dumping sites for dredged material in the North Sea. Environ Pollut. 2003;124(1):17–31. 10.1016/S0269-7491(02)00430-X. 12683979

[25] Boyd SE, Limpenny DS, Rees HL, Cooper KM. The effects of marine sand and gravel extraction on the macrobenthos at a commercial dredging site (results 6 years post-dredging). ICES J Mar Sci. 2005;62(2):145–62. 10.1016/j.icesjms.2004.11.014

[26] Hinchey E, Schaffner L, Hoar C, Vogt B, Batte L. Responses of Estuarine Benthic Invertebrates to Sediment Burial: The Importance of Mobility and Adaptation. Hydrobiologia. 2006;556(1):85–98. 10.1007/s10750-005-1029-0

[27] Rogers CS. Responses of coral reef organisms to sedimentation. Mar Ecol Prog Ser. 1990;62:185–202.

[28] Maurer D, Keck RT, Tinsman JC, Leathem WA. Vertical migration and mortality of benthos in dredged material—part I: Mollusca. Mar Environ Res. 1981;4(4):299–319. 10.1016/0141-1136(81)90043-X.

[29] Hendrick VJ, Hutchison ZL, Last K. Sediment burial intolerance of marine macroinvertebrates. PLOS ONE. Accepted. 10.1371/journal.pone.0149114 PMC4765823 26901775

[30] Kranz PM. The anastrophic burial of bivalves and it paeleoecologcial significance. J Geol. 1974;82(2):237–65.

[31] Fariñas-Franco JM, Sanderson WG, Roberts D. Phenotypic differences may limit the potential for habitat restoration involving species translocation: a case study of shape ecophenotypes in different populations of *Modiolus modiolus* (Mollusca: Bivalvia). Aquat Conserv: Mar Freshwat Ecosyst. 2014:n/a-n/a. 10.1002/aqc.2496

[32] Dinesen GE, Morton B. Review of the functional morphology, biology, perturbation impacts on the boreal, habitat forming horse mussel *Modiolus modiolus* (Bivalvia: Mytilida: Modiolinae). Mar Biol Res. 2014;10(9):845–70.

[33] Tyler-Walters H. *Mytilus edulis*. Common mussel Marine Life Information Network: Biology and Sensitivity Key Information Sub-programme [online]. Plymouth: Marine Biological Association of the United Kingdom; 2008 [2015]. Available: http://www.marlin.ac.uk/taxonomyidentification.php?speciesID=3848.

[34] Daly MA, Mathieson AC. The effects of sand movement on intertidal seaweeds and selected invertebrates at Bound Rock, New Hampshire, USA. Mar Biol. 1977;43(1):45–55. 10.1007/bf00392570

[35] Holt TJ, Rees EI, Hawkins SJ, Seed R. Biogenic Reefs (volume IX) An overview of dynamic and sensitivity characteristics for conservation management of marine SACs. Scottish Association for Marine Science (SAMS), 1998.

[36] Widdows J, Lucas JS, Brinsley MD, Salkeld PN, Staff FJ. Investigation of the effects of current velocity on mussel feeding and mussel bed stability using an annular flume. Helgol Mar Res. 2002;56(1):3–12. 10.1007/s10152-001-0100-0

[37] Tyler-Walters H. *Modiolus modiolus*. Horse Mussel Marine Life Information Network: Biology and Sensitivity Key Information Sub-programme [online] Plymouth: Marine Biological Association of the United Kingdom; 2007 [2015]. Available: http://www.marlin.ac.uk/speciessensitivity.php?speciesID=3817.

[38] Seed R, Suchaneck TH. Population and community ecology of *Mytilus edulis* In: Gosling EM, editor. The mussel Mytilus: ecology, physiology, genetics and culture. Development in Aquaculture and Fisheries Science. Amsterdam: Elsevier Science Publications; 1992 p. 87–169.

[39] Strong JA, Service M. Historical chronologies of sedimentation and heavy-metal contamination in Strangford Lough, Northern Ireland. Biology and Environment: Proceedings of the Royal Irish Academy. 2008;108B(2):109–26.

[40] Roberts D, Allock L, Fariñas-Franco JM, Gorman E, Maggs CA, Mahon AM, et al Modiolus Restoration Research Project: Final Report and Recommendations Queens University Belfast, Departments of Agriculture and Rural Development, and Northern Ireland Environment Agency, 2011.

[41] Farinas-Franco JM, Pearce B, Porter J, Harries D, Mair JM, Woolmer AS, et al Marine Strategy Framework Directive Indicators for Biogenic Reefs formed by *Modiolus modiolus*, *Mytius edulis* and *Sabellaria spinulosa* Part 1: Defining and validating the indicators Heriot Watt University for JNCC, JNCC Peterborough, 2014.

[42] Connor DW, Allen JH, Golding N, Howell KL, Lieberknecht LM, Northen KO, et al The marine habitat classification for Britain and Ireland Peterborough: Joint Nature Conservation Committee, 2004.

[43] Rees I. Assessment of *Modiolus modiolus* beds in the OSPAR area. Prepared by University of Wales, Bangor, UK, on behalf of the Joint Nature Conservation Committee (JNCC), 2008 revised in January 2009. Report No.

[44] Jackson JBC. What was natural in the coastal oceans? Proceedings of the National Academy of Sciences. 2001;98(10):5411–8. 10.1073/pnas.091092898 PMC33227 11344287

[45] van Duren LA, Herman PMJ, Sandee AJJ, Heip CHR. Effects of mussel filtering activity on boundary layer structure. J Sea Res. 2006;55:3–14.

[46] van Leeuwen B, Augustijin DCM, van Wesenbeeck BK, Hulscher S, de Vries MB. Modelling the influence of a young mussel bed on fine sediment dynamics on an intertidal flat in the Wadden Sea. Ecol Eng. 2010;36(145–153).

[47] Council Directive 92/43/EEC of 21 May 1992 on the conservation of natural habitats and of wild fauna and flora, (1992).

[48] Hendrick VJ, Foster-Smith RL, Davies AJ. Biogenic reefs and the marine aggregate industry (No. 3). Monograph. 2011.

[49] FAO. Fisheries and Aquaculture Information and Statistics Service 13/02/2014: Food and Agriculture Organisation; 2014 [cited 2014]. Available: http://www.fao.org/figis/servlet/SQServlet?ds=Aquaculture&k1=SPECIES&k1v=1&k1s=2688&outtype=html.

[50] The Crown Estate, Marine Resource System (MaRS), cartographers. All Offshore Activity (UK). London2014.

[51] Davies AJ, Last KS, Attard K, Hendrick VJ. Maintaining turbidity and current flow in laboratory aquarium studies, a case study using *Sabellaria spinulosa*. J Exp Mar Biol Ecol. 2009;370(1–2):35–40. 10.1016/j.jembe.2008.11.015 .

[52] Boyd SE, Limpenny DS, Rees HL, Cooper KM, Campbell S. Preliminary observations of the effects of dredging intensity on the re-colonisation of dredged sediments off the southeast coast of England (Area 222). Estuar Coast Shelf Sci. 2003;57(1–2):209–23. 10.1016/S0272-7714(02)00346-3.

[53] Chapple JP, Smerdon GR, Berry RJ, Hawkins AJS. Seasonal changes in stress-70 protein levels reflect thermal tolerance in the marine bivalve *Mytilus edulis* L. J Exp Mar Biol Ecol. 1998;229(1):53–68. 10.1016/S0022-0981(98)00040-9.

[54] Read KRH, Cumming KB. Thermal tolerance of the bivalve molluscs *Modiolus modiolus* L., *Mytilus edulis* L. and *Brachidontes demissus* Dillwyn. Comp Biochem Phys. 1967;22(1):149–55. 10.1016/0010-406X(67)90176-4. 6049983

[55] Babarro JM, Reiriz MJ. Secretion of byssal threads in *Mytilus galloprovincialis*: quantitative and qualitative values after spawning stress. J Comp Physiol B. 2010;180(1):95–104. 10.1007/s00360-009-0392-y 19618191

[56] Crosby MP, Gale LD. A review and evaluation of bivalve condition index methodologies with a suggested standard method. J Shellfish Res. 1990;9(1):233–7.

[57] R Core Team. R: A language and environment for statistical computing. Vienna, Austria: 2014.

[58] Ginzburg LR, Jensen CXJ. Rules of thumb for judging ecological theories. Trends Ecol Evol. 2004;19(3):121–6. 10.1016/j.tree.2003.11.004. 16701242

[59] Burnham KP, Anderson DR. Model selection and mutitmodel inference: a practical information-theoretic approach 2nd ed: Springer; 2002. 488 p.

[60] Cottrell RS, Black KD, Hutchison ZL, Last KS. The Influence of Organic Material and Temperature on the Burial Tolerance of the Blue Mussel, *Mytilus edulis*: Considerations for the Management of Marine Aggregate Dredging. PLOS ONE. 2016;11(1):e0147534 10.1371/journal.pone.0147534 26809153 PMC4726446

[61] Young GA. Byssus-thread formation by the mussel *Mytilus edulis*: effects of environmental factors. Mar Ecol Prog Ser. 1985;24:261–71.

[62] Meadows PS, Shand P. Experimental analysis of byssus thread production by *Mytilus edulis* and *Modiolus modiolus* in sediments. Mar Biol. 1989;101(2):219–26. 10.1007/bf00391461

[63] Pearce T, LaBarbera M. A comparative study of the mechanical properties of Mytilid byssal threads. J Exp Biol. 2009;212(10):1442–8. 19411537 10.1242/jeb.025544

[64] Brazee SL, Carrington E. Interspecific Comparison of the Mechanical Properties of Mussel Byssus. Biol Bull. 2006;211(3):263–74. 17179385 10.2307/4134548

[65] Pearce T, LaBarbera M. Biomechanics of byssal threads outside the Mytilidae: *Atrina rigida* and *Ctenoides mitis*. J Exp Biol. 2009;212(10):1449–54. 19411538 10.1242/jeb.025551

[66] Witman JD, Suchanek TH. Mussels in flow: drag and dislodgement by epizoans. Mar Ecol Prog Ser. 1984;16:259–68. citeulike-article-id:12594761 10.3354/meps016259

[67] Okamura B. Group Living and the Effects of Spatial Position in Aggregations of *Mytilus edulis*. Oecologia. 1986;69(3):341–7. 28311334 10.1007/BF00377054

[68] Trueman ER, Brand AR, Davis P. The dynamics of burrowing of some common littoral bivalves. J Exp Biol. 1966;44:469–92.

[69] Trueman ER, Brand AR, Davis P. The effect of substrate and shell shape on the burrowing of some common bivalves. Proc Malacol Soc Lond. 1966;37:97–109.

[70] Gosling EM. Chapter 7: Circulation, Respiration, Excretion and Osmoregulation Bivalve molluscs: biology, ecology and culture: Fishing New Books, Blackwell Publishing; 2003.

[71] Diaz RJ, Rosenberg R. Marine benthic hypoxia: A review of its ecological effects and the behavioural responses of benthic macrofauna. Oceanogr Mar Biol Annu Rev. 1995;33:245–303.

[72] Theede H, Ponat A, Hiroki K, Schlieper C. Studies on the resistance of marine bottom invertebrates to oxygen-deficiency and hydrogen sulphide. Mar Biol. 1969;2(4):325–37. 10.1007/bf00355712

[73] Newell RIE. Species Profiles: Life Histories and Environmental Requirements of Coastal Fisheries and Invertebrates (North and Mid-Atlantic); Blue Mussel. Horn Point Environmental Laboratories, University of Maryland, 1989 Contract No.: Report 82 (11.102), TR EL-82-4.

[74] Famme P, Knudsen J, Hansen ES. The effect of oxygen on the aerobic-anaerobic metabolism of the marine bivalve *Mytilus edulis*. Mar Biol Lett. 1981;2:345–51.

[75] Zandee DI, Howkwerda DA, Kluytmans JH, Zwaan AD. Metabolic adaptations to environmental anoxia in the intertidal bivalve mollusc *Mytilus edulis* L. Neth J Zool. 1986;36(3):322–43.

[76] Jørgensen BB. Seasonal Oxygen Depletion in the Bottom Waters of a Danish Fjord and Its Effect on the Benthic Community. Oikos. 1980;34(1):68–76. 10.2307/3544551

[77] Theede H. Comparative studies on the influence of oxygen deficiency and hydrogen sulphide on marine bottom invertebrates. Neth J Sea Res. 1973;7(0):244–52. 10.1016/0077-7579(73)90048-3.

[78] Wang WX, Widdows J. Physiological responses of mussel larvae *Mytilus edulis* to environmental hypoxia and anoxia. Mar Ecol Prog Ser. 1991;70:223–1991.

[79] Gormley KSG, Porter JS, Bell MC, Hull AD, Sanderson WG. Predictive Habitat Modelling as a Tool to Assess the Change in Distribution and Extent of an OSPAR Priority Habitat under an Increased Ocean Temperature Scenario: Consequences for Marine Protected Area Networks and Management. PLoS ONE. 2013;8(7):e68263 10.1371/journal.pone.0068263 23894298 PMC3718827

[80] Bayne B. Aspects of the metabolism of *Mytilus edulis* during starvation. Neth J Sea Res. 1973;7:399–410. 10.1016/0077-7579(73)90061-6.

[81] Riisgård HS, Egede PP, Saavedra IB. Feeding Behaviour of the Mussel, *Mytilus edulis*: New Observations, with a Minireview of Current Knowledge. J Mar Biol. 2011;Article ID: 312459:13 pages

[82] de Vooys CGN. Elimination of sand in the blue mussel, *Mytilus edulis*. Neth J Sea Res. 1987;21(1):75–8. 10.1016/0077-7579(87)90023-8

[83] Owen SF. Meeting energy budgets by modulation of behaviour and physiology in the eel (*Anguilla anguilla* L.). Comp Biochem Phys A. 2001;128(3):629–42. 10.1016/S1095-6433(00)00340-8. 11246050

[84] Widdows J, Hawkins AJS. Partitioning of Rate of Heat Dissipation by *Mytilus edulis* into Maintenance, Feeding, and Growth Components. Physiol Zool. 1989;62(3):764–84.

[85] Price HA. An Analysis of Factors Determining Seasonal Variation in the Byssal Attachment Strength of *Mytilus edulis*. J Mar Biol Assoc UK. 1982;62(01):147–55. 10.1017/S0025315400020178

[86] Bayne BL, Widdows J. The physiological ecology of two populations of *Mytilus edulis* L. Oecologia. 1978;37(2):137–62. 10.1007/bf00344987 28309646

[87] Dolmer P. The interactions between bed structure of *Mytilus edulis* L. and the predator *Asterias rubens* L. J Exp Mar Biol Ecol. 1998;228(1):137–50. 10.1016/s0022-0981(98)00024-0

