# Supplementary material for: Buried Alive: The Behavioural Response of the Mussels, Modiolus modiolus and Mytilus edulis to Sudden Burial by Sediment
Source: PLoS One. 2016 Mar 16;11(3):e0151471. doi: 10.1371/journal.pone.0151471 (PMC4794176; doi:10.1371/journal.pone.0151471)
Supplement: S1 Table — Results of the best fit binomial GLM for the probability of mortality in Modiolus modiolus (a) and Mytilus edulis (b) whilst under burial of variable durations (2, 4, 8, 16, 32 days), sediment fractions (coarse, medium and fine) and depths (2 cm, 5 cm, 7 cm). (DOCX) [file pone.0151471.s001.docx]

| **S1 Table.** Experiment 1. | | | | |
| --- | --- | --- | --- | --- |
| ***Modiolus modiolus*** | | | | |
| **Variable** | **Estimate** | **Std. Error** | **z value** | **p-value** |
| (Intercept) | -5.92 | 1.91 | -3.101 | **0.00193** |
| Log_10_Duration | 3.98 | 1.49 | 2.669 | **0.00760** |
| Fine Sediment | -71.12 | 8488.72 | -0.008 | 0.99332 |
| Medium Sediment | -7.58 | 5.07 | -1.494 | 0.23524 |
| Log_10_Duration:Fine Sediment | 60.58 | 7049.73 | 0.009 | 0.99314 |
| Log_10_Duration:Medium Sediment | 6.51 | 3.97 | 1.641 | 0.10087 |
| **AIC** | 73.06 |  |  |  |
| **Residual deviance** | 61.06 on 156 degrees of freedom | | |  |
| ***Mytilus edulis*** | | | | |
| **Variable** | **Estimate** | **Std. Error** | **z value** | **p-value** |
| (Intercept) | -9.35 | 1.84 | -5.089 | **3.60 e-07** |
| Log_10_Duration | 4.61 | 1.09 | 4.228 | **2.35e-05** |
| Fine Sediment | 3.94 | 1.16 | 3.403 | **0.000667** |
| Medium Sediment | 1.91 | 1.16 | 1.641 | 0.100731 |
| **AIC** | 75.89 |  |  |  |
| **Residual deviance** | 67.89 on 156 degrees of freedom | | |  |
