# Supplementary material for: Buried Alive: The Behavioural Response of the Mussels, Modiolus modiolus and Mytilus edulis to Sudden Burial by Sediment
Source: PLoS One. 2016 Mar 16;11(3):e0151471. doi: 10.1371/journal.pone.0151471 (PMC4794176; doi:10.1371/journal.pone.0151471)
Supplement: S2 Table — Results of the best fit binomial GLM for the probability of mortality in Mytilus edulis whilst under fine sediment burial for variable depths (2 cm, 5 cm, 7 cm), durations (16, 32 days) of burial and variable water temperatures (cold (8°C), ambient (14.5°C) and warm (20°C) water). (DOCX) [file pone.0151471.s002.docx]

| **S2 Table.** Experiment 2. | | | | |
| --- | --- | --- | --- | --- |
| **Variable** | **Estimate** | **Std. Error** | **z-value** | **p-value** |
| (Intercept) | -83.06 | 13245.17 | -0.006 | 0.9950 |
| Log_10_Duration | 69.02 | 10999.87 | 0.006 | 0.9950 |
| Cold water | -13.76 | 21417.08 | -0.001 | 0.9995 |
| Warm water | 85.50 | 13245.17 | 0.006 | 0.9948 |
| 5 cm Depth | -0.41 | 1.00 | -0.496 | 0.6201 |
| 2 cm Depth | -2.28 | 1.08 | -2.111 | **0.0348** |
| Log_10_Duration: Cold Water | -4.29 | 15685.32 | 0.000 | 0.9998 |
| Log_10_Duration: Warm Water | -69.02 | 10999.88 | -0.006 | 0.9950 |
| **AIC** | 52.91 |  |  |  |
| **Residual deviance** | 36.91 on 46 degrees of freedom | | |  |
