# Supplementary material for: Buried Alive: The Behavioural Response of the Mussels, Modiolus modiolus and Mytilus edulis to Sudden Burial by Sediment
Source: PLoS One. 2016 Mar 16;11(3):e0151471. doi: 10.1371/journal.pone.0151471 (PMC4794176; doi:10.1371/journal.pone.0151471)
Supplement: S3 Table — Results of the best fit binomial GLM for the probability of Mytilus edulis emerging from shallow, coarse sediment burial over increasing durations (a). It was confirmed that there was a higher level of byssus production in mussels that had emerged from burial (b). The use of the sediment, and hard surface in horizontal orientation was assessed and vertical surfaces were found to be important in the emergence behaviour (c). (DOCX) [file pone.0151471.s003.docx]

| **S3 Table.** Experiment 3b. | | | | |
| --- | --- | --- | --- | --- |
| ***Mytilus edulis* emergence over increasing duration of burial** | | | | |
| **Variable** | **Estimate** | **Std. Error** | **z-value** | **p-value** |
| (Intercept) | -0.80 | 0.40 | -2.016 | **0.043774** |
| Log_10_Duration | 2.44 | 0.64 | 3.877 | **0.000106** |
| **AIC** | 103.61 |  |  |  |
| **Residual deviance** | 99.61 on 88 degrees of freedom | | | |
| ***Mytilus edulis* byssus production** | | | | |
| **Variable** | **Estimate** | **Std. Error** | **z-value** | **p-value** |
| (Intercept) | -1.32 | 0.63 | -2.092 | **0.0364** |
| Post-burial Total byssus | 0.10 | 0.05 | 2.044 | **0.0410** |
| **AIC** | 47.78 |  |  |  |
| **Residual deviance** | 43.78 on 34 degrees of freedom | | | |
| ***Mytilus edulis* byssus production on variable orientations of hard surface** | | | | |
| **Variable** | **Estimate** | **Std. Error** | **z-value** | **p-value** |
| (Intercept) | -2.33 | 0.75 | -3.095 | **0.00197** |
| Vertical relief | 1.00 | 0.31 | 3.220 | **0.00128** |
| **AIC** | 23.60 |  |  |  |
| **Residual deviance** | 19.60 on 34 degrees of freedom | | | |
| ***Mytilus edulis* byssus production, pre- and post- burial** | | | | |
| **Variable** | **Estimate** | **Std. Error** | **z-value** | **p-value** |
| (Intercept) | -3.17 | 1.14 | -2.780 | **0.00544** |
| Pre-burial byssus | 0.10 | 0.09 | 1.166 | 0.24373 |
| Post-burial byssus | 0.33 | 0.10 | 3.245 | **0.00117** |
| Pre-burial byssus* Post-burial byssus | -0.01 | 0.01 | -2.129 | **0.03327** |
| **AIC** | 57.08 |  |  |  |
| **Residual deviance** | 49.08 on 50 degrees of freedom | | | |
